# Challenges in the care of patients with RET-altered thyroid cancer: a multicountry mixed-methods study

**DOI:** 10.1186/s13044-023-00166-4

**Published:** 2023-08-14

**Authors:** Suzanne Murray, Vivek Subbiah, Steven I. Sherman, Sophie Péloquin, Anthony Sireci, Christian Grohé, Patrick Bubach, Patrice Lazure

**Affiliations:** 1 AXDEV Group Inc, 210-8, Place du Commerce, Brossard, Québec J4W 3H2 Canada; 2 The University of Texas MD Anderson Cancer Center, 1515 Holcombe Blvd, Houston, TX 77030 USA; 3 Eli Lilly, 450 E 29th St 12th Floor, New York, NY 10016 USA; 4 Berlin Evangelical Lung Clinic, Lindenberger Weg 27, 13125 Berlin, Germany

**Keywords:** RET Gene Mutation, Thyroid Cancer, Delivery of Health Care, Medical oncology, Endocrinology, Pathology

## Abstract

**Background:**

The discovery of driver oncogenes for thyroid carcinomas and the identification of genomically targeted therapies to inhibit those oncogenes have altered the treatment algorithm in thyroid cancer (TC), while germline testing for RET mutations has become indicated for patients with a family history of RET gene mutations or hereditary medullary TC (MTC). In the context of an increasing number of selective RET inhibitors approved for use, this paper aims to describe challenges and barriers affecting providers’ ability to deliver optimal care for patients with RET-altered TC across the patient healthcare journey.

**Methods:**

A mixed-method educational and behavioral needs assessment was conducted in Germany (GER), Japan (JPN), the United Kingdom (UK), and the United States (US) prior to RET-selective inhibitor approval. Participants included medical oncologists (MO), endocrinologists (EN) and clinical pathologists (CP) caring for patients affected with TC. Data collection tools were implemented in three languages (English, German, Japanese). Qualitative data were coded and thematically analyzed in NVivo. Quantitative data were analyzed via frequency and crosstabulations in SPSS. The findings presented here were part of a broader study that also investigated lung cancer challenges and included pulmonologists.

**Results:**

A total of 44 interviews and 378 surveys were completed. Suboptimal knowledge and skills were self-identified among providers, affecting (1) assessment of genetic risk factors (56%, 159/285 of MOs and ENs), (2) selection of appropriate genetic biomarkers (59%, 53/90 of CPs), (3) treatment plan initiation (65%, 173/275 of MOs and ENs), (4) management of side effects associated with multitargeted tyrosine kinase inhibitors (78%, 116/149 of MOs and ENs), and (5) transfer of patients into palliative care services (58%, 160/274 of MOs and ENs). Interviews underscored the presence of systemic barriers affecting the use of RET molecular tests and selective inhibitors, in addition to suboptimal knowledge and skills necessary to manage the safety and efficacy of targeted therapies.

**Conclusion:**

This study describes concrete educational needs for providers involved in the care of patients with RET-altered thyroid carcinomas. Findings can be used to inform the design of evidence-based education and performance improvement interventions in the field and support integration into practice of newly approved RET-selective inhibitors.

## Background

A number of genetic and epigenetic studies have been completed in the last three decades to understand the pathogenesis of thyroid carcinomas [1]. The discovery of driver oncogenes and the identification of genomically targeted therapies to inhibit those oncogenes have altered the treatment algorithm for TC. One of the most important regulators of the mitogen active kinase (MAPK) signaling pathway in both medullary and papillary TCs is a receptor-tyrosine kinase encoded by the ‘rearranged during transfection’ (RET) gene [2]. Activation of this receptor triggers a cascade of events involved in cell growth, proliferation, and survival [3]. Approximately 50% of sporadic medullary TC (MTC) cases, and virtually all hereditary MTC cases, are associated with mutations in the RET gene [4]. Chimeric products resulting from fusion of RET kinase with other genes have also been identified and can vary by country depending on multiple factors, including ethnicity and exposure to radiation [5]. For example, RET/PTC fusions have been identified in 8% of PTC cases in Germany [6], compared with 30% in Japan [7], and may increase to up to 60–80% in areas exposed to radiation [8, 9].

The European Society of Medical Oncology (ESMO) and National Comprehensive Cancer Network (NCCN) suggest that germline testing for RET mutations is indicated for patients with a family history of RET gene mutations or hereditary MTC, patients with clinical features suspicious for multiple endocrine neoplasia type II, and newly diagnosed patients with clinically apparent sporadic MTC. ESMO recommends detecting RET rearrangements in nonmedullary thyroid carcinomas through next generation sequencing techniques or fluorescent in situ hybridization (FISH) when sufficient tissue is provided and the risk of RET abnormality is high [10]. Analysis of RET mutations and fusion can be accomplished via commercially available DNA or RNA next generation sequencing (NGS) assays, including multianalyte assays that also assess other targetable alterations [10].

Understanding the molecular pathologies associated with TC has greatly impacted the development of new targeted therapies, with an increasing number of selective RET inhibitors demonstrating promising results compared with multikinase inhibitors (MKIs) [11, 12]. Recently, selpercatinib and prasletinib were granted approval from the Food and Drug Administration (FDA), and selpercatinib was granted approval from the Committee for Medicinal Products for Human Use (CHMP) of the European Medicines Agency (EMA) for the treatment of patients with advanced or metastatic RET-mutant MTC or radioactive iodine-refractory RET fusion-positive TC patients [13–15].

As the field of precision medicine in TC continues to grow, healthcare professionals (HCPs) are expected to stay abreast of evolving scientific advancements regarding new targeted therapies and associated genomic tests. A crucial step in bridging the gap between current and best practice is to assess the educational needs of HCPs across this expanding continuum of patient care [16].

The study objectives were (1) to report on the healthcare journey of patients with RET-altered TC (HCPs involved, services received, and transfer in care between providers) and (2) to identify challenges and barriers experienced by HCPs in the care of patients with RET-driven TC. Similar objectives were established in relation to the care of RET-altered lung cancer (LC) patients, which is being reported separately.

## Methods

This study employed a parallel mixed-methods design with qualitative semi-structured interviews and a quantitative online survey. Interviews documented the current practices, challenges, and barriers to optimal care. The survey assessed the magnitude and frequency of these practices, barriers, and challenges [17]. Both interview and survey questions assessed self-reported knowledge, skills, attitude, confidence, and systemic or contextual barriers (e.g., access to resources) [18, 19]. Triangulation of data sources, methods, and perspectives was performed [20].

### Ethical approval

The study was approved by an independent ethics review board (VERITAS IRB, Quebec, Canada).

### Selection and description of participants

Two physician databases operating in compliance with the guidelines of the European Society for Opinion and Marketing Research were used to recruit potential participants [21]. Email invitations included a secured URL to an online screener. Inclusion criteria were: practicing in Germany (GER), Japan (JPN), the UK, or the US; either (a) medical/clinical oncologist with a minimum of 20 TC and 20 LC patients per year, (b) endocrinologist with a minimum of 10 TC patients per year, or (c) pathologist analyzing a minimum of 10 TC and 10 LC samples per year; and three years of practice or more; minimum of 50% time spent caring for patients. Data were monitored to ensure that a diverse sample of participants was obtained (e.g., mix of regions within each country) via purposive sampling [22].

### Data collection

Interview guides and surveys were developed in English based on a literature review and discussion with subject matter experts (SMEs; i.e., co-authors VS, SIS, KN, AS, and CG) [23]. Data collection tools were adapted for each specialty’s scope of practice. Semi-structured interviews (45 min) included 18–22 open-ended questions with suggested probes to elicit comprehensive responses [24]. The 31-item survey (20 min) asked participants to rate their perceived level of knowledge and skill (5-point rating scale), confidence (100-point visual analogue rating scale), or agreement (5-point Likert scale) with various items [25, 26]. The option of selecting “not relevant to my current role” was provided to ensure ratings accounted for the perceived roles and responsibilities of participants. In addition, participants were asked to select one or more response that best described their approach to RET-altered TC patients [27]. Data collection tools were translated into German and Japanese.

A briefing session was held between researchers and interviewers to ensure alignment with the intent of the interview questions and probes [28]. Interviews were conducted in the participants’ language of choice over a secure call. Upon participant consent, audio was recorded, transcribed, and translated to English when required. Surveys were programmed on a secured webpage and tested for accuracy and navigation experience.

### Analysis & statistics

#### Qualitative analysis

A coding tree was developed *a priori* in NVivo 12 (QSR International Pty Ltd.) to categorize relevant transcript information by key area of exploration [29–31]. Researchers coded transcripts and regularly discussed required modifications to the coding tree based on emerging themes. Thematic analysis was performed to identify trends in reported experiences and perspectives by country and specialty [31]. Visual maps were created through an iterative process to depict patients’ healthcare journey.

#### Quantitative analysis

Values representing knowledge and skill ratings were dichotomized as follows: 1 (none), 2 (basic), and 3 (intermediate) were grouped as ‘suboptimal’; 4 (advanced) and 5 (expert) were grouped as ‘optimal’. Values representing agreement ratings were regrouped as follows: 1 (strongly disagree) with 2 (disagree); 3 (neither agree nor disagree) unchanged; 4 (agree) with 5 (strongly agree). Frequency tables were run for demographic variables. Differences by country and specialty were analyzed via crosstabulations with chi-square statistics. Non-parametric Kruskal H Wallis tests were performed on confidence rating variables to assess differences in mean rankings between country and specialty [32]. Missing values and data from participants who selected “not relevant to my current role” were excluded from the analysis for each specific question. All statistical analyses were performed using IBM SPSS Statistics (Version 26.0. Armonk, NY: IBM Corp.)

#### Triangulation

Findings from both qualitative and quantitative phases were compared to identify areas of convergence [20, 33]. The findings were interpreted with the expertise of clinical SMEs and adult education specialists (co-authors SM, SP, PL) to provide context on the reported patient healthcare journey in each country and identify the most pressing educational needs for each specialty [16, 20].

## Results

**Fig. 1:**
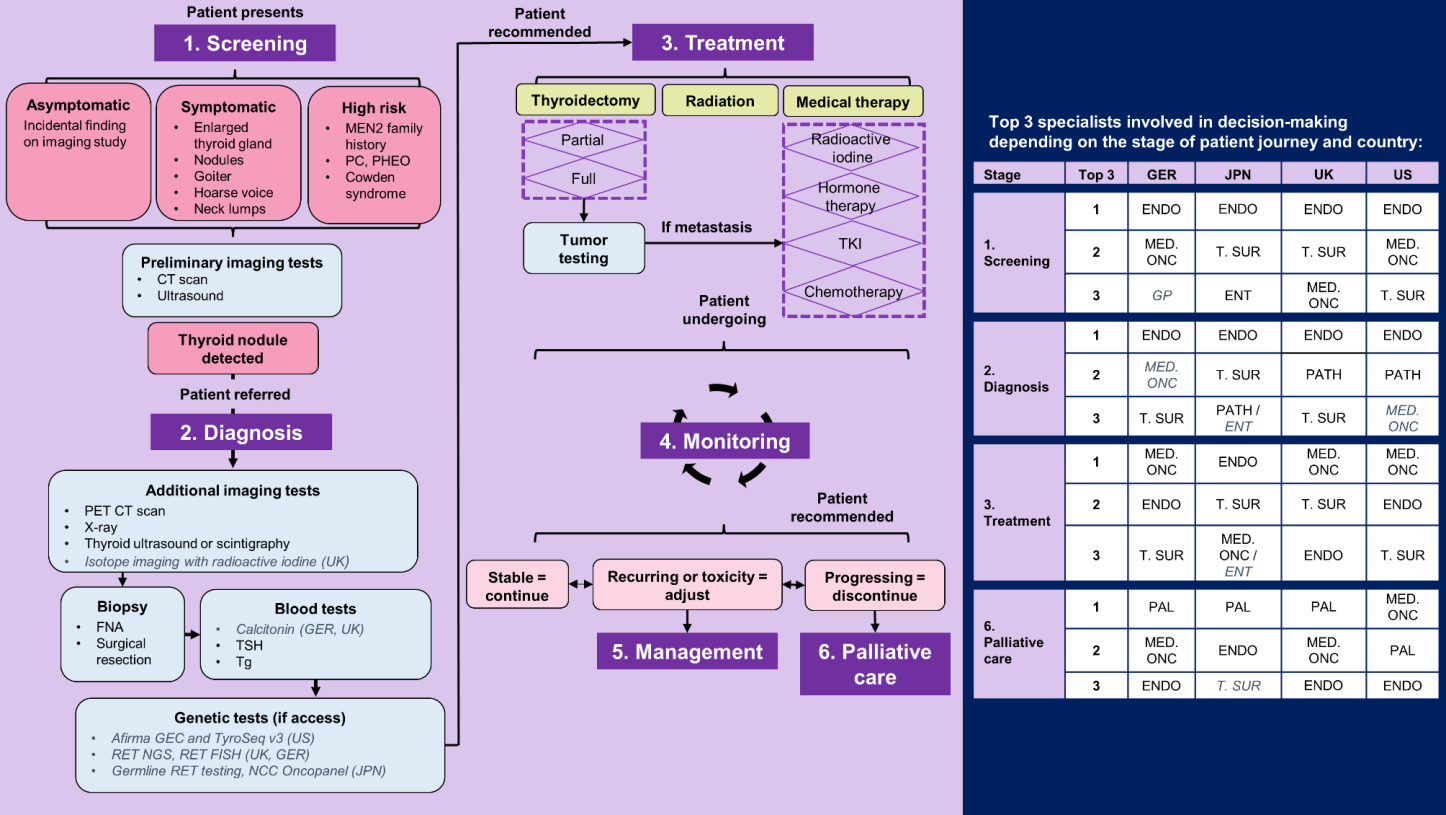
**Overview of the healthcare journey of patients with RET-altered thyroid cancer** *Details*: Services provided during screening, diagnosis, treatment, monitoring, management and palliative care. Differences by country are demonstrated in italics. The top 3 specialists involved at each stage of the patient journey are reported on the right *Legend*: MEN2 = Multiple endocrine neoplasia type 2, PC = parathyroid carcinoma, PHEO = phleochromocytoma, FNA = fine needle aspiration, TSH = thyroid stimulating hormone, Tg = thyroglobulin, NGS = next generation sequencing, FISH = fluorescence in situ hybridization, DTC = differentiated thyroid carcinoma such as papillary or follicular thyroid carcinoma, TKI = tyrosine kinase inhibitor, ENDO = endocrinologist, MED. ONC = medical oncologist, T. SUR = thyroid surgeon, GP = general practitioner or primary care physician, ENT = otorhinolaryngologist or ear-nose-throat specialist, PATH = pathologist, PAL = palliative care physician, GER = Germany, UK = United Kingdom, JPN = Japan, US = United States.

A total of 422 participants completed the study (44 interviews and 378 surveys). A similar demographic representation was obtained for both phases (Table 1), and a lot of variation was reported by participants in terms of thyroid cancer caseload (Table 1). Triangulated findings pertaining to the healthcare journey of patients with RET-altered TC (Fig. 1) alongside challenges and barriers across the continuum of care include (1) screening, (2) diagnosis, (3) treatment, (4) monitoring and management, and (5) palliative care.

**Table 1:** Descriptive statistics by study phase.

		Semi-structured interviews (n = 44)		Online survey (n = 378)		Total (n = 422)	
		**%**	*n*	**%**	*n*	**%**	*n*
**Country**	Germany	**27**	*12*	**24**	*90*	**24**	*102*
	United Kingdom	**18**	*8*	**25**	*96*	**25**	*104*
	Japan	**27**	*12*	**19**	*73*	**20**	*85*
	United States	**27**	*12*	**32**	*119*	**31**	*131*
**Specialty**	Medical oncologists	**36**	*12*	**35**	*133*	**34**	*145*
	Endocrinologists	**27**	*16*	**41**	*154*	**40**	*170*
	Pathologists	**36**	*16*	**24**	*91*	**25**	*107*
**Years of practice**	3–10 years	**56**	*7*	**25**	*96*	**24**	*103*
	11–20 years	**27**	*25*	**51**	*191*	**51**	*216*
	21 years or more	**16**	*12*	**21**	*91*	**24**	*103*
**Practice setting**	NCCN-affiliated / NCI-designated cancer center	**0**	*0*	**1**	*3*	**1**	*3*
	Specialized cancer center	**23**	*10*	**6**	*21*	**7**	*31*
	Academic hospital	**39**	*17*	**47**	*178*	**46**	*195*
	Community hospital	**16**	*7*	**14**	*52*	**14**	*59*
	Community clinic	**5**	*2*	**1**	*4*	**1**	*6*
	Multi-specialty physician group practice	**7**	*3*	**18**	*66*	**16**	*69*
	Single-specialty physician group practice	**5**	*2*	**9**	*33*	**8**	*35*
	Solo practice	**7**	*3*	**5**	*18*	**5**	*21*
	Government medicine (e.g. Veterans Affairs)	**0**	*0*	**0.3**	*1*	**0.2**	*1*
	Other	**0**	*0*	**0.5**	*2*	**0.5**	*2*
**Academic affiliation**	Yes (practice setting is academic affiliated)	**61**	*17*	**54**	*202*	**52**	*219*
	No (practice setting is community based)	**39**	*27*	**46**	*174*	**48**	*201*

Table shows sample demographics obtained from the qualitative phase (semistructured interviews) and quantitative phase (online survey). Significant differences were found in years of practice and academic affiliation depending on country in the quantitative phase sample (p < 0.05): 3–10 years (13% in Germany, 24% in the United Kingdom, 26% in Japan, 35% in the United States), 11–20 years (70% in Germany, 45% in the United Kingdom, 44% in Japan, 46% in the United States), 21 years or more (17% in Germany, 31% in the United Kingdom, 30% in Japan, 20% in the United States), and academic affiliation (56% in Germany, 83% in the United Kingdom, 55% in Japan and 28% in the United States).

Bold for % and italics for n were used to increase legibility of the table.

**Table 2:** Participants’ reported caseload for thyroid cancer.

		Semi-structured interviews (n = 44)			Online survey (n = 378)		
		**Mean**	*Median*	Range	**Mean**	*Median*	Range
**Specialty**	Medical oncologists* (n = 12, 133)	**48**	*30*	20–180	**177**	*78*	*20-1800*
	Endocrinologists* (n = 16, 154)	**191**	*100*	12–600	**69**	*50*	*10–500*
	Pathologists** (n-16, 91)	**275**	*50*	10-2000	**159**	*80*	*10-2000*

* Medical oncologists and Endocrinologists were asked “What is your caseload of thyroid cancer patients per year?”

** Pathologists were asked “How many samples per year do you analyze (from biopsy/surgery) to inform the diagnostic or treatment of thyroid cancer?”.

Bold for means and italics for medians were used to increase legibility of the table.

### Screening

Interviewees reported that the healthcare journey of patients with RET-altered TC begins when patients present to an endocrinologist, medical oncologist, thyroid surgeon, or general practitioner (in GER), with symptoms (e.g., neck lumps) or high risk of TC (e.g., family history of multiple endocrine neoplasia type 2 or MEN2). Patients then undergo preliminary imaging, neck ultrasound or CT scans. Alternatively, asymptomatic patients are identified through incidental imaging.

… if you have a known gene mutation which is related, let’s say, to a condition called Cowden syndrome, then you have an increased risk of thyroid cancer, and those patients would have screening. So, it would only be for select groups.

-Endocrinologist, UK.

Barriers to optimal care at this stage include suboptimal knowledge of screening tools (40%, 113/282) and genetic risk factors of TC (56%, 159/285) found among both medical oncologists and endocrinologists with statistically significant differences (p < 0.05) by country (Table 3).

**Table 3:** Percent of providers reporting suboptimal knowledge in the care of patients with RET-altered thyroid cancer.

		GER		UK		JPN		US		Total		
Sub-optimal knowledge of:	Profession	%	*n*	%	*n*	%	*n*	%	*n*	%	*n*	Sig.
**Screening tools for TC**	MED. ONC	**57**	*44*	**37**	*41*	**-**	*-*	**36**	*44*	**43**	*129*	0.087
	ENDO	**35**	*26*	**27**	*41*	**67**	*43*	**19**	*43*	**37**	*153*	> 0.001*
	PATH	**-**	*-*	**-**	*-*	**-**	*-*	**-**	*-*	**-**	*-*	-
**Genetic risk factors of TC**	MED. ONC	**68**	*44*	**56**	*43*	**-**	*-*	**39**	*44*	**54**	*131*	0.020*
	ENDO	**54**	*26*	**51**	*41*	**78**	*43*	**46**	*44*	**57**	*154*	0.019*
	PATH	**-**	*-*	**-**	*-*	**-**	*-*	**-**	*-*	**-**	*-*	-
**Genetic biomarker tests for TC**	MED. ONC	**52**	*44*	**52**	*44*	**-**	*-*	**44**	*45*	**50**	*133*	0.694
	ENDO	**50**	*26*	**48**	*41*	**77**	*43*	**39**	*44*	**62**	*154*	> 0.001*
	PATH	**30**	*20*	**73**	*11*	**79**	*29*	**53**	*30*	**59**	*90*	0.004*
**Tests required in order to initiate treatment for TC**	MED. ONC	**55**	*44*	**40**	*43*	**-**	*-*	**42**	*45*	**46**	*132*	0.322
	ENDO	**54**	*26*	**42**	*41*	**62**	*42*	**25**	*44*	**44**	*153*	0.005*
	PATH	**30**	*20*	**73**	*11*	**76**	*29*	**70**	*30*	**63**	*90*	0.006*
**Specific mechanisms of action of multi-kinase inhibitors for TC**	MED. ONC	**50**	*44*	**41**	*44*	**-**	*-*	**42**	*45*	**44**	*133*	0.650
	ENDO	**58**	*26*	**73**	*40*	**69**	*42*	**76**	*41*	**70**	*149*	0.455
	PATH	**40**	*20*	**82**	*11*	**83**	*29*	**72**	*29*	**70**	*89*	0.009*
**Specific mechanisms of action of selective RET inhibitors for TC**	MED. ONC	**61**	*44*	**47**	*44*	**-**	*-*	**44**	*45*	**54**	*133*	0.252
	ENDO	**68**	*25*	**83**	*40*	**46**	*41*	**77**	*39*	**77**	*145*	0.608
	PATH	**50**	*20*	**75**	*8*	**78**	*27*	**83**	*29*	**73**	*84*	0.071
**National guidelines for the management of TC**	MED. ONC	**41**	*44*	**32**	*44*	**-**	*-*	**38**	*45*	**37**	*133*	0.668
	ENDO	**42**	*26*	**37**	*41*	**56**	*43*	**33**	*43*	**42**	*153*	0.142
	PATH	**35**	*20*	**82**	*11*	**84**	*30*	**72**	*29*	**69**	*90*	0.002*
**International guidelines for the management of TC**	MED. ONC	**39**	*44*	**50**	*44*	**-**	*-*	**49**	*45*	**46**	*133*	0.498
	ENDO	**46**	*26*	**44**	*41*	**69**	*43*	**58**	*43*	**56**	*153*	0.078
	PATH	**55**	*20*	**91**	*11*	**97**	*30*	**83**	*29*	**82**	*18*	0.002*
**Side effects of multi-kinase inhibitors**	MED. ONC	**46**	*44*	**34**	*44*	**-**	*-*	**39**	*44*	**39**	*132*	0.547
	ENDO	**77**	*26*	**80**	*40*	**79**	*42*	**76**	*41*	**78**	*149*	0.969
	PATH	**-**	*-*	**-**	*-*	**-**	*-*	**-**	*-*	**-**	*-*	-
**Side-effects of selective RET inhibitors**	MED. ONC	**64**	*44*	**52**	*44*	**-**	*-*	**41**	*44*	**52**	*132*	0.103
	ENDO	**80**	*25*	**88**	*40*	**85**	*41*	**77**	*39*	**83**	*145*	0.596
	PATH	**-**	*-*	**-**	*-*	**-**	*-*	**-**	*-*	**-**	*-*	-
**Ongoing clinical trials on selective RET inhibitors**	MED. ONC	**67**	*43*	**73**	*44*	**-**	*-*	**61**	*44*	**67**	*131*	0.525
	ENDO	**87**	*23*	**90**	*40*	**90**	*40*	**85**	*39*	**88**	*142*	0.861
	PATH	**45**	*20*	**86**	*7*	**86**	*27*	**90**	*29*	**77**	*82*	0.002*

GER = Germany, UK = United Kingdom, JPN = Japan, US = United States.

* significant difference between countries (p<0.05) bold for % and italics for n were used to increase legibility of the table.

### Diagnosis

During interviews, participants described how patients with thyroid nodules are diagnosed by an endocrinologist, medical oncologist, or thyroid surgeon with the help of pathologists. Diagnostic modalities include ultrasound, PET CT scan, X-ray, thyroid scintigraphy, isotope imaging with radioactive iodine (UK), followed by fine needle aspiration (FNA) or surgical resection, as well as blood tests of calcitonin (GER, UK), thyroid stimulating hormone (TSH) or thyroglobulin (Tg) levels. If accessible, RET testing is performed via FISH (specifically for fusions) or NGS (for fusions and/or mutations). Access to these tests depends on laboratory resources, patient insurance, and physicians’ understanding of the diagnostic and prognostic significance of available biomarkers for various forms of TC.

Since they thought that the patient had papillary cancer, they didn’t think that a genetic test was necessary, and after the surgery was done, they realized that the patient had medullary cancer—that was scary …

-Pathologist, Japan.

Sub-optimal knowledge of genetic biomarker tests for TC was reported by 50% of medical oncologists (66/133), 62% of endocrinologists (95/154) and 59% of pathologists (53/90) (Table 3). In addition, over three-fifths of endocrinologists (63%, 96/153) reported sub-optimal skills determining if a genetic biomarker test is necessary to inform the diagnosis, and 59% of pathologists (53/90) reported sub-optimal skills selecting the appropriate genetic biomarker(s) to diagnose TC (Table 4).

**Table 4:** Percent of providers reporting suboptimal skill in the care of patients with RET-altered thyroid cancer.

		GER		UK		JPN		US		Total			
Sub-optimal skill in:	Profession	%	*n*	%	*n*	%	*n*	%	*n*	%	*n*	Sig.	
**Deciding which genetic biomarker test(s) to order when TC is suspected**	MED. ONC	**55**	*44*	**48**	*44*	**-**	*-*	**33**	*45*	**45**	*133*	0.121	
	ENDO	**62**	*26*	**73**	*41*	**77**	*43*	**41**	*44*	**63**	*154*	0.002*	
	PATH	**-**	*-*	**-**	*-*	**-**	*-*	**-**	*-*	**-**	*-*	-	
**Determining if a genetic biomarker test is necessary to inform the diagnosis**	MED. ONC	**-**	*-*	**-**	*-*	**-**	*-*	**-**	*-*	**-**	*-*	-	
	ENDO	**58**	*26*	**73**	*41*	**74**	*43*	**44**	*43*	**63**	*153*	0.012*	
	PATH	**-**	*-*	**-**	*-*	**-**	*-*	**-**	*-*	**-**	*-*	-	
**Selecting the appropriate genetic biomarker(s) to diagnose TC**	MED. ONC	**-**	*-*	**-**	*-*	**-**	*-*	**-**	*-*	**-**	*-*	-	
	ENDO	**-**	*-*	**-**	*-*	**-**	*-*	**-**	*-*	**-**	*-*	-	
	PATH	**25**	*20*	**73**	*11*	**23**	*29*	**57**	*30*	**59**	*90*	0.001*	
**Determining the initial treatment plan after staging RET-altered TC**	MED. ONC	**52**	*44*	**52**	*44*	**-**	*-*	**36**	*45*	**47**	*133*	0.188	
	ENDO	**75**	*24*	**85**	*39*	**85**	*41*	**82**	*38*	**82**	*142*	0.728	
	PATH	**-**	*-*	**-**	*-*	**-**	*-*	**-**	*-*	**-**	*-*	-	
**Managing side effects of multi-kinase inhibitors**	MED. ONC	**41**	*44*	**27**	*44*	**-**	*-*	**36**	*44*	**35**	*132*	0.393	
	ENDO	**80**	*25*	**85**	*39*	**81**	*42*	**84**	*38*	**83**	*144*	0.945	
	PATH	**-**	*-*	**-**	*-*	**-**	*-*	**-**	*-*	**-**	*-*	-	
**Managing side effects of selective RET inhibitors**	MED. ONC	**64**	*44*	**57**	*44*	**-**	*-*	**39**	*44*	**53**	*132*	0.052	
	ENDO	**79**	*24*	**85**	*39*	**85**	*41*	**84**	*37*	**84**	*141*	0.926	
	PATH	**-**	*-*	**-**	*-*	**-**	*-*	**-**	*-*	**-**	*-*	-	
**Identifying eligible patients for clinical trials of selective RET inhibitors**	MED. ONC	**60**	*42*	**46**	*44*	**-**	*-*	**34**	*44*	**46**	*130*	0.061	
	ENDO	**79**	*24*	**87**	*39*	**88**	*40*	**79**	*39*	**84**	*141*	0.624	
	PATH	**-**	*-*	**-**	*-*	**-**	*-*	**-**	*-*	**-**	*-*	-	
**Identifying the signs of TC progression**	MED. ONC	**46**	*44*	**21**	*44*	**-**	*-*	**31**	*45*	**32**	*133*	0.042	
	ENDO	**84**	*26*	**37**	*41*	**69**	*42*	**35**	*43*	**48**	*152*	0.005*	
	PATH	**35**	*20*	**90**	*10*	**87**	*29*	**74**	*27*	**71**	*86*	0.001*	
**Identifying the relevant genetic biomarker(s) to inform the progression of TC**	MED. ONC	**52**	*44*	**55**	*44*	**-**	*-*	**37**	*45*	**47**	*133*	0.146	
	ENDO	**69**	*26*	**69**	*39*	**81**	*42*	**48**	*44*	**66**	*151*	0.011*	
	PATH	**40**	*20*	**73**	*11*	**83**	*29*	**63**	*30*	**66**	*90*	0.019*	
**Determining when the initial treatment plan should be changed due to RET-altered TC progression**	MED. ONC	**64**	*44*	**52**	*44*	**-**	*-*	**42**	*45*	**53**	*133*	0.129	
	ENDO	**75**	*24*	**92**	*38*	**85**	*41*	**87**	*39*	**86**	*142*	0.305	
	PATH	**-**	*-*	**-**	*-*	**-**	*-*	**-**	*-*	**-**	*-*	-	
**Determining when palliative care is appropriate for a patient with thyroid RET cancer**	MED. ONC	**57**	*44*	**30**	*43*	**-**	*-*	**33**	*45*	**40**	*132*	0.021*	
	ENDO	**75**	*24*	**67**	*40*	**85**	*40*	**74**	*38*	**75**	*142*	0.335	
	PATH	**-**	*-*	**-**	*-*	**-**	*-*	**-**	*-*	**-**	*-*	-	

MED. ONC = Medical Oncologist, ENDO = Endocrinologist, PATH = Pathologist

GER = Germany, UK = United Kingdom, JPN = Japan, US = United States.

* significant difference between countries (p<0.05) bold for % and italics for n were used to increase legibility of the table.

### Treatment

Barriers to assessing the molecular profile of RET-altered TC patients include suboptimal skills among medical oncologists and endocrinologists (55%, 157/287) in deciding which genetic biomarker test to order and suboptimal skills among pathologists (59%, 53/90) in selecting appropriate genetic biomarkers with statistically significant differences (p < 0.05) by country (Table 3).

Interviewees described how patients with operable TC undergo thyroidectomy. If the disease progresses, radiation therapy and/or systemic therapy (i.e., radioactive iodine, hormone therapy, immunotherapy, MKIs) is administered. Selective RET inhibitors were reported as only administered in the scope of clinical trials. Treatment decisions are based on tumor histology and stage. Patient age, comorbidities, existing medications, health status, and insurance coverage are also considered. Experienced medical oncologists may discuss off-label treatments with their patients.

A challenge in planning and determining treatment was identified. According to survey data, 65% (173/275) of endocrinologists and medical oncologists reported suboptimal skills determining the initial treatment plan after staging of RET-altered TC. The mean confidence score for determining the treatment plan in a patient with RET-altered TC was 51%.

**Fig. 2:**
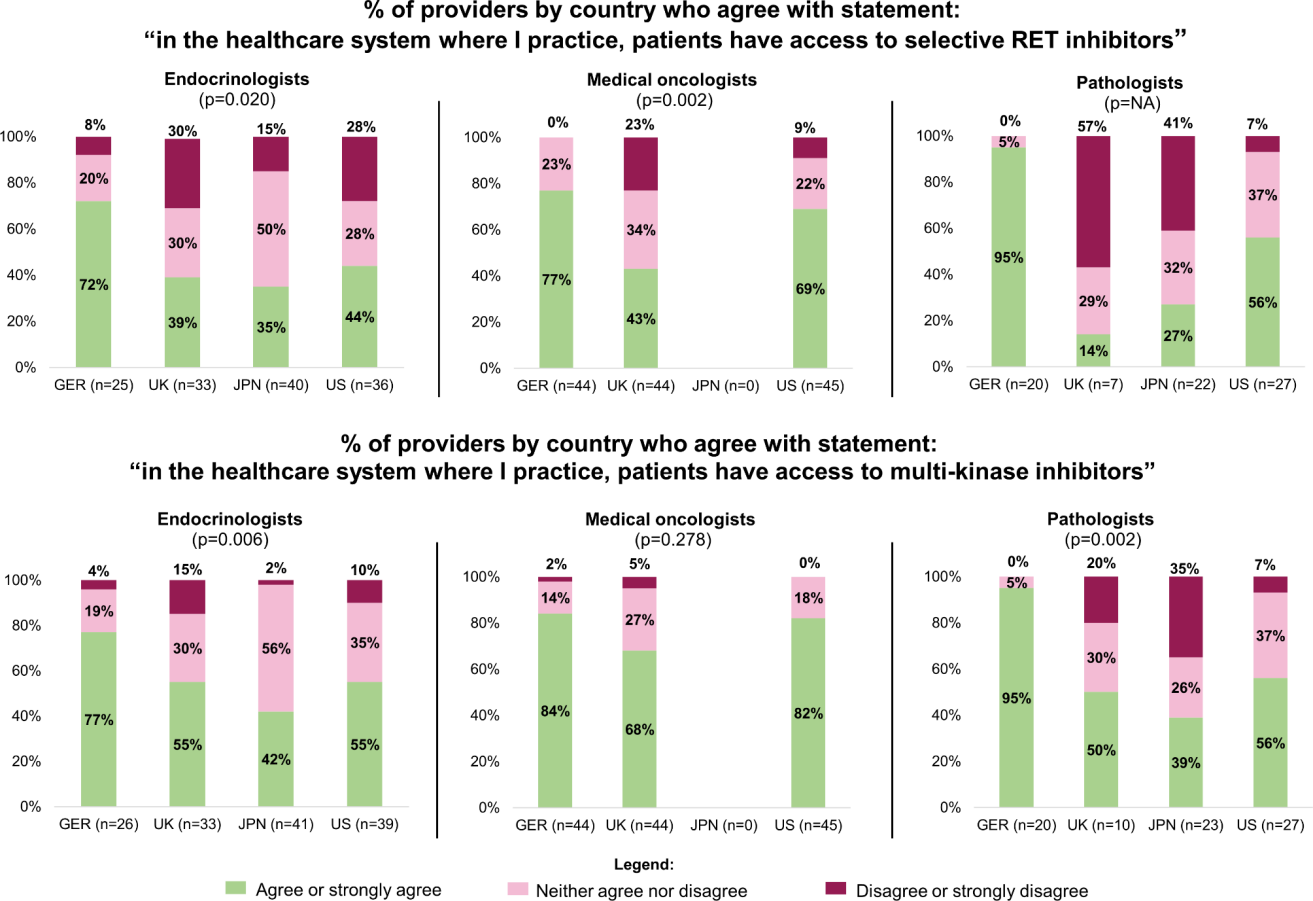
**Percent of providers by country who agree with statement regarding access to treatment** *Details*: Significance of differences by country of each profession (endocrinologist, medical oncologist, or pathologist) indicated by p-value in parentheses below title *Legend*: Dark pink indicates % who responded “disagree or strongly disagree”, light pink “neither agree nor disagree”, green “agree or strongly agree” GER = Germany, UK = United Kingdom, JPN = Japan, US = United States.

Statistically significant differences were found among countries in providers’ perspectives regarding patient access to RET-selective inhibitors and multikinase inhibitors (Fig. 2).

### Monitoring and management

Based on interview data, treatment adjustments are made for patients on an ongoing basis (e.g., when disease recurs or an adverse event is reported). In this process, many providers associated a high rate of side effects with new medications for RET fusion mutations and were concerned about their ability to manage these side effects, especially in an outpatient setting. On average, surveyed endocrinologists reported suboptimal knowledge of side effects associated with MKIs (78% (116/149)) and selective RET inhibitors (83% (120/145)), compared to 40% (52/132) and 52% (69/132), respectively, for medical oncologists. Interviewees erroneously perceived that patients on selective RET inhibitors would likely require more dose adjustment due to side effects.

… the RET specific TKIs have a really high-level toxicity so that approximately half the patients need at least a dose reduction because of the side effects.

-Endocrinologist, Germany

Over two-thirds (70%; 192/275) of endocrinologists and medical oncologists reported suboptimal skills determining when the initial treatment plan should be changed due to RET-altered TC progression.

### Palliative Care

Interviewees described that some RET-altered TC patients experience critical disease progression causing severe declines in quality of life. For these patients, palliative care may be suggested, resulting in referral to palliative care specialists. A suboptimal level of skill was reported by 58% (160/274) of endocrinologists and medical oncologists in determining when this service is appropriate, with a statistically significant difference by country among medical oncologists (Table 3).

## Discussion

This study provides a clearer picture of the healthcare journey of patients with RET-altered TC and a better understanding of the HCPs involved, services received, and how care transfers between providers. The findings suggest the need to improve medical oncologists’, endocrinologists’, and clinical pathologists’ knowledge of the predictive value of RET testing in TC. The need to improve all specialists’ skill and confidence when selecting germline or somatic RET testing for patients with inherited or sporadic MTC, respectively, was identified. Although selpercatinib and prasletinib were not approved by any national regulatory bodies for the treatment of TC patients at the time of data collection, these findings suggest suboptimal knowledge among medical oncologists regarding ongoing clinical trials on selective RET inhibitors. Furthermore, medical oncologists and endocrinologists experience challenges managing the side effects of selective RET inhibitors (especially in an outpatient setting), likely due to suboptimal knowledge of potential side effects and toxicity management skills.

This study identified gaps in knowledge of screening tools and in the skills to determine which genetic biomarker tests to order, selecting appropriate genetic biomarkers and determining treatment plans. Current guidelines detail recommendations for HCPs in this area, suggesting that additional CME is needed to support the integration of available knowledge into practice [10, 34].

There were gaps in the skills and knowledge needed to support optimal decision-making at key points in patient care. HCPs reported a misplaced perception that frequent treatment changes are needed due to adverse events associated with selective RET inhibitors. This suggests that HCPs may not be aware of current studies that show an improved safety profile among emerging treatments and may therefore base consequential treatment decisions on outdated information. Similarly, challenges were reported when making changes to the treatment plan due to RET-altered TC progression and determining when to suggest palliative care. Considering new studies on treatment for patients with advanced TC and the widespread gaps in TC quality of life considerations [35], these gaps may have an impact on the patient’s health outcomes and experience of care [36]. To improve in this area, HCPs could benefit from education designed to improve practical decision-making skills with a consideration of current and emerging treatment options [37]. The impact of these initiatives may be widespread: studies show that patients are more satisfied with their care when provided with education from informed caregivers [38].

### Implications for Clinicians and Policy-makers

The present findings can be used to develop continuous educational programs for HCPs involved in the diagnosis, treatment, and management of advanced TC patients. The behavioral change wheel may be used as a framework in linking the most appropriate intervention design to the educational needs identified in this study [39, 40]. For instance, online lectures could be delivered by experts in precision medicine to build and reinforce the knowledge base of medical oncologists and endocrinologists in relation to available biomarker tests and targeted therapies for patients with MTC or PTC [41]. A suggested emphasis may be placed on the relevance of RET testing (e.g., FISH or fusion testing, germline for mutations, tumor NGS for mutations or fusions) and ongoing clinical trials for selective RET inhibitors in addition to registry data providing real-word evidence on the safety and efficacy of available TKIs [42]. A decision-making tool to assist clinicians in the identification and referral of eligible TC patients with RET alterations to existing clinical trials could prove useful [43]. A patient-friendly tool could be developed to inform patients of available clinical trials for which they may be eligible [44]. Case-based learning opportunities may support skill and confidence acquisition among medical oncologists and allied HCPs in managing side-effects associated with selective RET inhibitors and other types of TKIs [45, 46].

Policymakers should consider optimizing reimbursement and payment models to encourage adherence to guidelines for the screening, diagnosis, treatment, and management of RET-altered TC patients. There is an opportunity for guidelines to be updated regularly to capture the rapid pace of testing and treatment advancements for patients with RET-altered TC.

### Strengths

The mixed-methods approach leveraged the strengths of qualitative (collecting rich, contextual information) and quantitative (assessing frequency and magnitude, comparison by demographics) research methods [17, 47]. Purposive sampling minimized the risk of selection bias by including a diverse representation of medical oncologists, endocrinologists, and clinical pathologists. A mix of years of practice, genders, regions within each country, thyroid cancer caseload and access to genomic testing was considered in the generation of findings. Data sources, methods, and perspectives were triangulated with current published evidence and guidelines during the interpretation and generation of final findings, thereby minimizing biases associated with single-observed and single-method studies.

### Limitations

The patient perspective was not included in the collection and analysis of data. The practices and competencies of providers were self-reported, which increases subjective reporting. Survey items were not validated for internal consistency reliability, short-term retest correlations, and convergent validity. However, they were critically reviewed by clinical SMEs and educational experts to optimize face validity, readability, comprehension, and relevancy within the clinical context. When interpreting findings, caution should be used when considering the applicability to countries, practice settings, and specialties excluded from this study.

### Recommendations for Future Research

Future studies may develop and evaluate interventions addressing the challenges identified by this study [16, 48, 49]. Implementation research should determine the best interventions to optimize care for patients with TC and/or validate the presence of suboptimal practices in RET-altered TC patient care via observational studies, assessment of patient registry data, or inclusion of patients in data collection and analysis [50–52]. Similar studies may investigate clinical practice gaps, challenges, and barriers experienced by stakeholders excluded from this study (e.g., thyroid surgeons).

## Conclusions

This mixed-methods study revealed the current healthcare journey of patients with RET-altered TC in Germany, Japan, the UK, and the US and the challenges and barriers experienced by medical oncologists, endocrinologists, and pathologists along the way. Educational needs were identified, including the needs to improve: knowledge of MTC and PTC risk and the value of RET molecular tests; skills assessing the efficacy versus toxicity profile of emerging targeted therapies in RET-altered tumors; and transitioning RET-altered TC patients into palliative care. Future interventions may provide needed support by addressing advancements in RET-altered TC care via online lecture-based and case-based learning.

## Data Availability

The full datasets used and/or analysed during the current study are available from the corresponding author on reasonable request.

## Abbreviations

CHMP: Committee for Medicinal Products for Human Use
CP: Clinical pathologist
EMA: European Medicines Agency
EN: endocrinologist
ESMO: The European Society of Medical Oncology
FISH: Fluorescent in situ hybridization
FNA: Fine needle aspiration
GER: Germany
HCP: Healthcare professional
JPN: Japan
LC: Lung cancer
MAPK: Mitogen-activated protein kinase
MEN2: Multiple endocrine neoplasia type 2
MKI: Multikinase inhibitor
MO: Medical oncologist
MTC: Medullary thyroid cancer
NCCN: National Comprehensive Cancer Network
NGS: Next generation sequencing
PTC: Papillary thyroid cancer
RET: Rearranged during transfection
TC: Thyroid cancer
Tg: Thyroglobulin
TKI: Tyrosine kinase inhibitor
TSH: Thyroid stimulating hormone
UK: United Kingdom
US: United States

## References

[1] Itchaki G, Brown JR (2018). Experience with ibrutinib for first-line use in patients with chronic lymphocytic leukemia. Ther Adv Hematol.

[2] Ishizaka Y, Itoh F, Tahira T, Ikeda I, Sugimura T, Tucker J (1989). Human ret proto-oncogene mapped to chromosome 10q11. 2. Oncogene.

[3] Arighi E, Borrello MG, Sariola H (2005). RET tyrosine kinase signaling in development and cancer. Cytokine Growth Factor Rev.

[4] Barletta JA, Nosé V, Sadow PM. Genomics and epigenomics of medullary thyroid carcinoma: from sporadic disease to familial manifestations. Endocr Pathol. 2021:1–9. 10.1007/s12022-021-09664-3 PMC9353617 33492588

[5] Khan MS, Qadri Q, Makhdoomi MJ, Wani MA, Malik AA, Niyaz M (2020). RET/PTC gene rearrangements in thyroid carcinogenesis: assessment and clinico-pathological correlations. Pathol Oncol Res.

[6] Mayr B, Pötter E, Goretzki P, Rüschoff J, Dietmaier W, Hoang-Vu C (1998). Expression of RET/PTC1,-2,-3,-∆3 and-4 in german papillary thyroid carcinoma. Br J Cancer.

[7] Wajjwalku W, Nakamura S, Hasegawa Y, Miyazaki K, Satoh Y, Funahashi H (1992). Low frequency of rearrangements of the ret and trk proto-oncogenes in japanese thyroid papillary carcinomas. Jpn J Cancer Res.

[8] Bounacer A, Wicker R, Schlumberger M, Sarasin A, Suarez H (1997). Oncogenic rearrangements of the ret proto-oncogene in thyroid tumors induced after exposure to ionizing radiation. Biochimie.

[9] Smida J, Salassidis K, Hieber L, Zitzelsberger H, Kellerer AM, Demidchik EP (1999). Distinct frequency of ret rearrangements in papillary thyroid carcinomas of children and adults from Belarus. Int J Cancer.

[10] Belli C, Penault-Llorca F, Ladanyi M, Normanno N, Scoazec J-Y, Lacroix L et al. ESMO recommendations on the standard methods to detect RET fusions and mutations in daily practice and clinical research. Ann Oncol. 2021. 10.1016/j.annonc.2020.11.021 33455880

[11] Drilon A, Hu ZI, Lai GG, Tan DS (2018). Targeting RET-driven cancers: lessons from evolving preclinical and clinical landscapes. Nat Reviews Clin Oncol.

[12] Subbiah V, Cote GJ (2020). Advances in targeting RET-dependent cancers. Cancer Discov.

[13] Markham A (2020). Selpercatinib: first approval. Drugs.

[14] Della Corte C, Morgillo F. Rethinking treatment for RET-altered lung and thyroid cancers: selpercatinib approval by the EMA. ESMO open. 2021;6(1). 10.1016/j.esmoop.2020.100041 PMC7820024 33477006

[15] US Food and Drug Administration. FDA approves pralsetinib for RET-altered thyroid cancers 2020 [updated 12/01/2020. Available from: https://www.fda.gov/drugs/drug-approvals-and-databases/fda-approves-pralsetinib-ret-altered-thyroid-cancers#:~:text=On%20December%201%2C%202020%2C%20the,fusion%2Dpositive%20thyroid%20cancer%20who.

[16] Moore DE (1998). Needs assessment in the new health care environment: combining discrepancy analysis and outcomes to create more effective CME. J Continuing Educ Health Professions.

[17] Creswell JW, Klassen AC, Plano Clark VL, Smith KC (2011). Best practices for mixed methods research in the health sciences.

[18] Atkins L, Francis J, Islam R, O’Connor D, Patey A, Ivers N (2017). A guide to using the theoretical domains Framework of behaviour change to investigate implementation problems. Implement Sci.

[19] West R, Michie S. A brief introduction to the COM-B model of behaviour and the PRIME theory of motivation [v1]. Qeios. 2020.

[20] Turner SF, Cardinal LB, Burton RM (2017). Research design for mixed methods: a triangulation-based framework and roadmap. Organizational Res Methods.

[21] ICC/ESOMAR. ICC/ESOMAR international code on market, opinion and social research and data analytics 2016 [Available from: https://www.esomar.org/what-we-do/code-guidelines].

[22] Etikan I, Musa SA, Alkassim RS (2016). Comparison of convenience sampling and purposive sampling. Am J theoretical Appl Stat.

[23] Kallio H, Pietilä AM, Johnson M, Kangasniemi M (2016). Systematic methodological review: developing a framework for a qualitative semi-structured interview guide. J Adv Nurs.

[24] Bearman M (2019). Eliciting rich data: a practical approach to writing semi-structured interview schedules. Focus on Health Professional Education: A Multi-disciplinary Journal.

[25] Simms LJ, Zelazny K, Williams TF, Bernstein L (2019). Does the number of response options matter? Psychometric perspectives using personality questionnaire data. Psychol Assess.

[26] Chyung SY, Roberts K, Swanson I, Hankinson A (2017). Evidence-based survey design: the use of a midpoint on the Likert scale. Perform Improv.

[27] Considine J, Botti M, Thomas S (2005). Design, format, validity and reliability of multiple choice questions for use in nursing research and education. Collegian.

[28] Wilson C. Interview techniques for UX practitioners: a user-centered design method. Morgan Kaufmann Publishers; 2013. [Available from: 10.1016/C2012-0-06209-6]

[29] O’Flaherty B, Whalley J. Qualitative analysis software applied to is research-Developing a coding strategy. ECIS 2004 Proceedings. 2004:123.

[30] Zamawe FC (2015). The implication of using NVivo software in qualitative data analysis: evidence-based reflections. Malawi Med J.

[31] Roberts K, Dowell A, Nie J-B (2019). Attempting rigour and replicability in thematic analysis of qualitative research data; a case study of codebook development. BMC Med Res Methodol.

[32] McKight PE, Najab J. Kruskal-wallis test. The corsini encyclopedia of psychology. 2010:1-.

[33] O’Cathain A, Murphy E, Nicholl J. Three techniques for integrating data in mixed methods studies. BMJ. 2010;341. 10.1136/bmj.c4587 20851841

[34] National Comprehensive Cancer Network. Thyroid Carcinoma Version 3.2021: National Comprensive Cancer Network (NCCN); 2021 [cited 2021 October 15]. Available from: https://www.nccn.org/professionals/physician_gls/pdf/thyroid.pdf.

[35] James BC, Aschebrook-Kilfoy B, White MG, Applewhite MK, Kaplan SP, Angelos P (2018). Quality of life in thyroid cancer—assessment of physician perceptions. J Surg Res.

[36] Salvatore D, Santoro M, Schlumberger M (2021). The importance of the RET gene in thyroid cancer and therapeutic implications. Nat Reviews Endocrinol.

[37] Shojaie D, Hoffman AS, Amaku R, Cabanillas ME, Sosa JA, Waguespack SG (2021). Decision making when Cancer becomes chronic: needs Assessment for a web-based medullary thyroid carcinoma patient decision aid. JMIR formative research.

[38] Díez JJ, Galofré JC (2021). Thyroid cancer patients satisfaction at the management outcome: an analysis of the results of a nationwide survey in 485 subjects. BMC Health Serv Res.

[39] Michie S, Atkins L, West R. The behaviour change wheel. A guide to designing interventions 1st ed Great Britain: Silverback Publishing. 2014:1003-10.

[40] Cowdell F, Dyson J (2019). How is the theoretical domains framework applied to developing health behaviour interventions? A systematic search and narrative synthesis. BMC Public Health.

[41] Roy M, Chen H, Sippel RS (2013). Current understanding and management of medullary thyroid cancer. Oncologist.

[42] Gautschi O, Milia J, Filleron T, Wolf J, Carbone DP, Owen D (2017). Targeting RET in patients with RET-rearranged lung cancers: results from the global, multicenter RET registry. J Clin Oncol.

[43] Tamborero D, Dienstmann R, Rachid MH, Boekel J, Baird R, Braña I (2020). Support systems to guide clinical decision-making in precision oncology: the Cancer Core Europe Molecular Tumor Board Portal. Nat Med.

[44] Fleisher L, Ruggieri DG, Miller SM, Manne S, Albrecht T, Buzaglo J (2014). Application of best practice approaches for designing decision support tools: the preparatory education about clinical trials (PRE-ACT) study. Patient Educ Couns.

[45] McLean SF (2016). Case-based learning and its application in medical and health-care fields: a review of worldwide literature. J Med Educ Curric Dev.

[46] Liu L, Li M, Zheng Q, Jiang H (2020). The Effects of Case-Based teaching in nursing Skill Education: cases do Matter. INQUIRY: The Journal of Health Care Organization Provision and Financing.

[47] O’cathain A, Murphy E, Nicholl J (2008). The quality of mixed methods studies in health services research. J Health Serv Res Policy.

[48] Moore GF, Audrey S, Barker M, Bond L, Bonell C, Hardeman W et al. Process evaluation of complex interventions: Medical Research Council guidance. BMJ. 2015;350. 10.1136/bmj.h1258 PMC4366184 25791983

[49] Moscoso SC, Chaves SS, Vidal MP, Argilaga MTA (2013). Reporting a program evaluation: needs, program plan, intervention, and decisions. Int J Clin Health Psychol.

[50] Dasch B, Kalies H, Feddersen B, Ruderer C, Hiddemann W, Bausewein C (2017). Care of cancer patients at the end of life in a german university hospital: a retrospective observational study from 2014. PLoS ONE.

[51] Hahlweg P, Didi S, Kriston L, Härter M, Nestoriuc Y, Scholl I (2017). Process quality of decision-making in multidisciplinary cancer team meetings: a structured observational study. BMC Cancer.

[52] LeBlanc TW, Abernethy AP (2017). Patient-reported outcomes in cancer care—hearing the patient voice at greater volume. Nat reviews Clin Oncol.

